# Evolution of a Bacterial Regulon Controlling Virulence and Mg^2+^ Homeostasis

**DOI:** 10.1371/journal.pgen.1000428

**Published:** 2009-03-20

**Authors:** J. Christian Perez, Dongwoo Shin, Igor Zwir, Tammy Latifi, Tricia J. Hadley, Eduardo A. Groisman

**Affiliations:** 1 Department of Molecular Microbiology, Washington University School of Medicine, St. Louis, Missouri, United States of America; 2 Howard Hughes Medical Institute, Washington University School of Medicine, St. Louis, Missouri, United States of America; Universidad de Sevilla, Spain

## Abstract

Related organisms typically rely on orthologous regulatory proteins to respond to a given signal. However, the extent to which (or even if) the targets of shared regulatory proteins are maintained across species has remained largely unknown. This question is of particular significance in bacteria due to the widespread effects of horizontal gene transfer. Here, we address this question by investigating the regulons controlled by the DNA-binding PhoP protein, which governs virulence and Mg^2+^ homeostasis in several bacterial species. We establish that the ancestral PhoP protein directs largely different gene sets in ten analyzed species of the family *Enterobacteriaceae*, reflecting both regulation of species-specific targets and transcriptional rewiring of shared genes. The two targets directly activated by PhoP in all ten species (the most distant of which diverged >200 million years ago), and coding for the most conserved proteins are the *phoPQ* operon itself and the lipoprotein-encoding *slyB* gene, which decreases PhoP protein activity. The Mg^2+^-responsive PhoP protein dictates expression of Mg^2+^ transporters and of enzymes that modify Mg^2+^-binding sites in the cell envelope in most analyzed species. In contrast to the core PhoP regulon, which determines the amount of active PhoP and copes with the low Mg^2+^ stress, the variable members of the regulon contribute species-specific traits, a property shared with regulons controlled by dissimilar regulatory proteins and responding to different signals.

## Author Summary

Organisms often respond to environmental cues by modifying the patterns of expression of multiple genes. Related species typically rely on orthologous DNA-binding regulatory proteins to orchestrate the response to a given stimulus. However, it is unclear whether different organisms express similar or rather distinct groups of genes in response to the same signal. This is of special interest in bacteria because even closely related species, such as *Escherichia coli* and *Salmonella enterica*, have quite distinct lifestyles and display significant differences in gene content. Here, we have addressed this question by investigating the sets of genes that the Mg^2+^-responding PhoP protein controls in ten enteric species. We established that the majority of targets governed by the PhoP protein are not shared across species. This is due to regulation of species-specific genes and also to transcriptional rewiring of shared genes. The few PhoP-regulated targets retained across species encode the most conserved proteins, which determine the level of active PhoP protein and mediate Mg^2+^ homeostasis. These findings demonstrate that self-governing the activity of a regulatory protein is critical for its proper functioning, even though it may regulate largely different gene sets across species.

## Introduction

The ability of an organism to orchestrate responses to environmental changes often depends on transcriptional regulatory proteins that control the expression of multiple genes. Related species typically rely on orthologous regulatory proteins to respond to a given stimulus. However, the extent to which the targets of regulation of such orthologs (*i.e.*, the regulon) are retained across species is not clear. This is of special interest in bacteria due to the rampant effects of horizontal gene transfer (reviewed in [1],[2]), which raises questions about how bacterial regulons have been shaped by widespread gains and subsequent losses of genes, and about the role played both by the conserved targets of regulation as well as by those that are species-specific. By contrast, in eukaryotes, where most of the experimental studies on the evolution of gene regulation have been carried out, transcriptional rewiring (*i.e.* gains and/or losses of interactions between orthologous regulatory proteins and orthologous target genes) appears to be the main source of variability in regulatory networks [3]–[5] as related eukaryotic species have similar gene content. We have addressed the evolution of bacterial gene regulation by investigating the regulons controlled by the DNA-binding regulatory protein PhoP in several enteric species, the most distant of which shared a common ancestor >200 million years ago.

The activity of the DNA-binding regulatory protein PhoP is dictated by its cognate sensor PhoQ, which responds to the extracytoplasmic levels of Mg^2+^: transcription of PhoP-activated genes is promoted in low Mg^2+^ and repressed in high Mg^2+^ [6]. The PhoP regulon has been best characterized in the human pathogen *Salmonella enterica* serovar Typhimurium, where the PhoP protein regulates ∼3% of the genes [7] both directly by binding to its target promoters, and indirectly by altering the levels and/or activity of other regulatory proteins and systems [8]. These PhoP-activated targets include Mg^2+^ transporters, enzymes involved in the covalent modification of cell envelope components, as well as virulence proteins whose biochemical activities remain largely undefined.

Despite establishing different interactions with their animal and plant hosts, the bubonic plague agent *Yersinia pestis*, the diarrhea-causing *Shigella flexneri* and the plant-pathogen *Erwinia carotovora* depend on a functional PhoP/PhoQ system to cause disease [9]–[14], like *Salmonella* [15]–[17]. The *phoPQ* genes are also found in the human commensal *Escherichia coli* as well as in the soil dwelling *Klebsiella pneumoniae* and in *Sodalis glossinidius*, a secondary symbiont of the tsetse fly. The presence of the PhoP/PhoQ system in this phenotypically diverse group of bacteria suggests that PhoP may regulate the expression of different sets of genes across species (*i.e.*, each regulon might be suited to the niche in which each organism proliferates.) Alternatively or in addition, PhoP could control cellular functions that are shared among different species in spite of their distinct lifestyles.

Here we examine the evolution of the PhoP regulon across ten species of the family *Enterobacteriaceae*. We experimentally determine that PhoP has adopted largely different targets of regulation, the result of horizontal gene transfer events that altered gene content even among closely related species. We show that transcriptional rewiring events involving gains and/or losses of interactions between PhoP and shared genes have shaped the PhoP regulon and may contribute to phenotypic differences between organisms. Moreover, we establish that the core members of the PhoP regulon (*i.e.*, those maintained in all analyzed members of the *Enterobacteriaceae* family) participate in regulatory loops designed to control the level and activity of the PhoP/PhoQ system; and that those PhoP-regulated target genes common to most species ensure Mg^2+^ homeostasis. This demonstrates that governing the activity of the PhoP/PhoQ regulatory system is critical for its proper functioning independently of how different its regulated targets might be.

## Results/Discussion

### Identifying Genes Directly Regulated by the PhoP Protein in *Y. pestis*

We sought to identify the PhoP-regulated genes in the plague agent *Y. pestis* because: First, a functional PhoP protein is necessary for virulence in mice and for survival inside mammalian cells [13], as in *Salmonella* [15]–[18]. And second, *S. enterica* and *Y. pestis* are distantly-related members of the family *Enterobacteriaceae*, having diverged from their last common ancestor >200 million years ago [19]. To enable the identification of genes regulated by the PhoP protein and to distinguish between genes that are directly and indirectly regulated by PhoP, we used both expression microarray analysis of wild-type vs. *phoP* mutant strains as well as chromatin immunoprecipitation followed by array hybridization (ChIP-chip) on custom-made whole genome tiling arrays (a *Y. pestis* strain harboring an epitope-tagged *phoP* gene in the chromosome was utilized for ChIP and as the wild-type strain in the expression microarrays.) Three biological replicates of each sample (RNA or DNA) were prepared from *Y. pestis* cells grown in defined medium containing low (50 µM) Mg^2+^, which are inducing conditions for the PhoP/PhoQ system, and hybridized to custom-designed NimbleGen tiling microarrays (Figure S1). Data from the six expression microarrays (three wild-type and three *phoP* ^−^) were combined for downstream data processing whereas the three ChIP-chip data sets were analyzed individually.

Based solely on the expression microarray data (*i.e.* without including the ChIP-chip results), 31 and 14 transcription units appeared to be activated and repressed by the *phoP* gene, respectively (Table S2) (see Text S1 for a description of how the tiling microarray data were processed and Table S1 for a list of all the probes that exhibited differential expression (>2-fold)). We note that a single transcription unit may entail several co-transcribed ORFs (which is readily inferred from the expression pattern of contiguous probes in the tiling array) as well as transcripts corresponding to genes not previously annotated. This set of transcripts represents potential direct and indirect targets of the PhoP protein.

We considered a transcription unit to be directly regulated by PhoP if there was a ChIP peak <300 bp from the 5′ end of a transcript that appeared *phoP*-regulated in the expression microarrays. In most cases there were ChIP peaks with significant scores (FDR≤0.05) in similar positions in all three biological replicates. We used real time PCR to verify whether these transcripts were indeed *phoP*-regulated and if the promoter regions of these transcripts were enriched in the ChIP samples (data not shown). Then, we identified their transcription start sites by primer extension or S1 mapping, and determined that these start sites were *phoP*-dependent. PhoP binding sites were experimentally and/or computationally identified within 100 nt upstream of the transcription start sites. The location and orientation of the PhoP boxes in all these promoters are similar to those in well-characterized PhoP-activated promoters in *E. coli* and *S. enterica* [7],[20]. Sixteen transcripts met all these criteria (Table S3). Thus, in this study we refer to these sixteen transcripts as the *Yersinia* PhoP regulon. (We found ChIP peaks in front of only two repressed transcripts (Table S2) suggesting that PhoP may repress them directly.)

A list of PhoP regulon members in *Y. pestis* biovar Microtus has been recently reported [21]. Curiously, only four of the 18 promoters described as directly regulated by PhoP overlap with the data set presented here. The reason(s) for the discrepancy are not entirely clear, but could be due to the following: First, our approach was based on a genome-wide search for *in vivo* PhoP binding regions located <300 bp away from *phoP*-dependent transcripts identified *in vivo* whereas Li. et al. [21] investigated binding of the purified PhoP protein *in vitro* to DNA fragments located upstream of ORFs that could be *phoP*-regulated. Second, Li et al. grew *Yersinia* under conditions [22] that are different from those shown to induce the *Yersinia* PhoP/PhoQ system effectively [23]. And third, the *Y. pestis* strain utilized by Li et al. is not the same as that examined in this work. Importantly, the structures of all the PhoP-regulated promoters identified in our study resemble those of well-characterized PhoP-activated promoters in other organisms [7],[24],[25]. By contrast, the only promoters described in [21] that harbor structures reminiscent of previously characterized PhoP-dependent promoters are those overlapping with our data, and the remaining putative PhoP-activated promoters contain PhoP boxes at locations uncharacteristic of a bacterial transcriptional activator (*i.e.*, >100 nt upstream of the transcription start site, overlapping with the −10 sequence, or positioned downstream of the transcription start site.)

### The PhoP Regulons of *Y. pestis* and *S. enterica*

**Figure 1:**
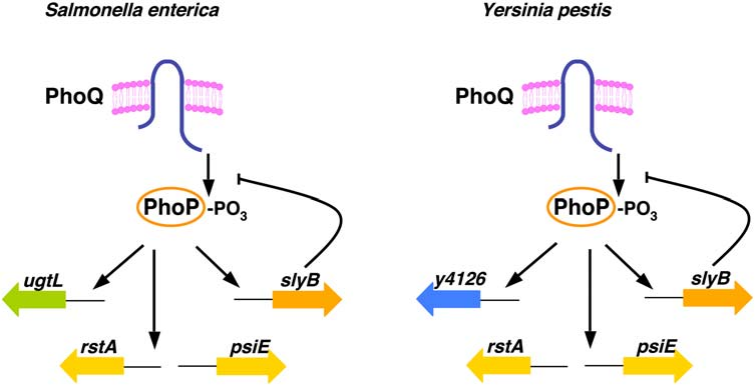
Diagram depicting examples of PhoP-regulated targets based on their occurrence in *S. enterica* and *Y. pestis.* The *Salmonella*-specific *ugtL* gene has no homolog in *Yersinia*, and the converse is true for the *Yersinia y4126* gene. As discussed in the main text, this class of genes is by far the most abundant and their sporadic distribution in other enteric species suggest that they may have been horizontally acquired. The *rstA* and *psiE* genes are present in both species but are controlled by PhoP in only one of the two species. Genes of this class (five out of sixteen) represent transcriptional rewiring events, a phenomenon that has not been experimentally addressed in bacteria. The *slyB* gene is controlled by PhoP in both *Yersinia* and *Salmonella*, and encodes a product that negatively regulates PhoP activity and is critical for the proper functioning of the PhoP/PhoQ system.

Based on their occurrence in *Y. pestis* and *S. enterica*, we classified the identified PhoP-regulated targets from *Yersinia* and the 20 transcription units known to be directly activated by PhoP in *S. enterica* [7],[24],[26] into three groups: (1) genes/operons present in *Yersinia* but not in *Salmonella*, or vice versa; (2) genes/operons present in both species but controlled by PhoP in only one of the two species; and (3) orthologous genes/operons controlled by PhoP in both *Yersinia* and *Salmonella* (Figure 1). As discussed below, the first group of genes is by far the most abundant and their sporadic distribution in other enteric species suggest that they have been horizontally acquired. The second group of genes (five out of sixteen) represent transcriptional rewiring events, a phenomenon that, to our knowledge, has not been experimentally addressed in bacteria. The third group of genes is the smallest set and, as the results presented in the following sections indicate, they appear to play distinct critical roles in the proper functioning of the PhoP/PhoQ system.

### Most genes Directly Regulated by PhoP in *Y. pestis* or in *S. enterica* Have No Homologs in Other Enteric Species

**Figure 2:**
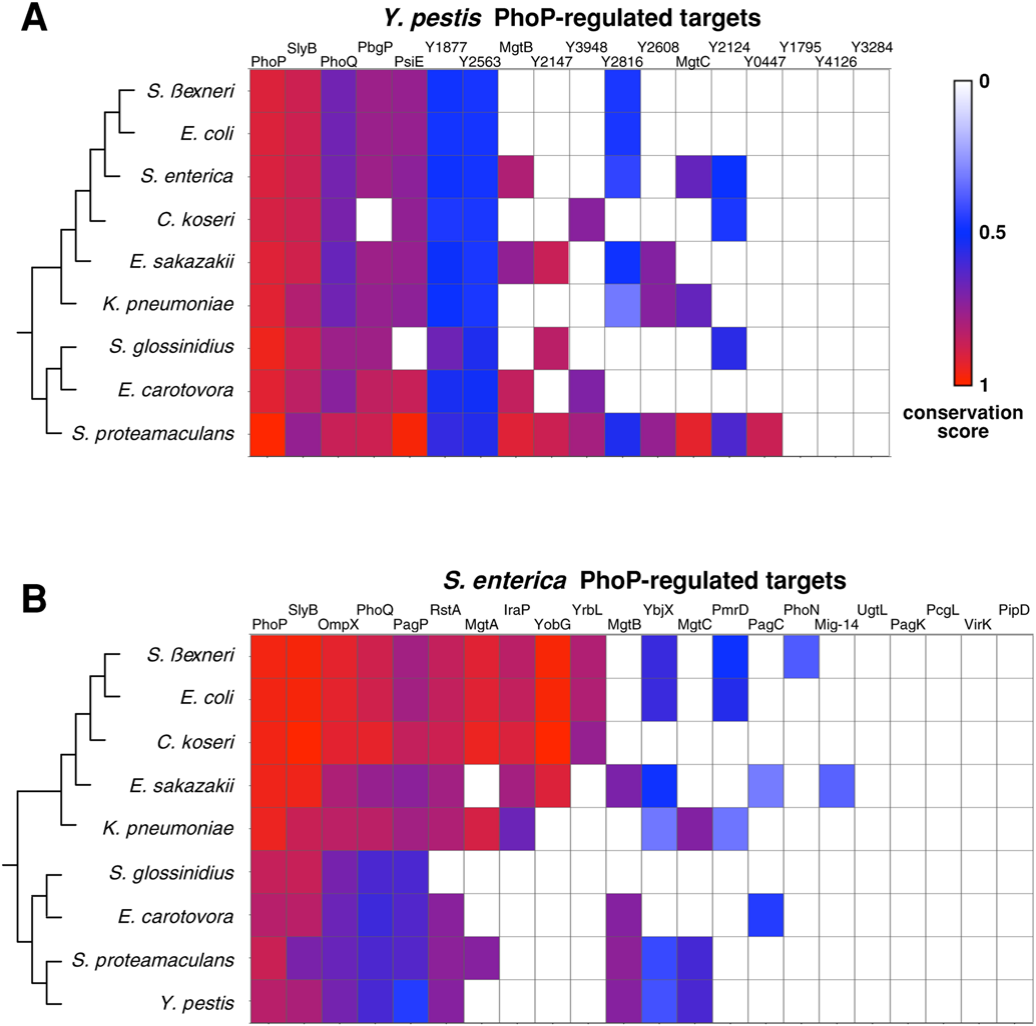
Limited distribution of *Y. pestis* and *S. enterica* PhoP-activated genes across the *Enterobacteriaceae* family. A–B. Matrices of conservation scores (CS) of proteins encoded by the genes directly activated by PhoP in *Y. pestis* (A) and *S. enterica* (B). CS is the BlastP score of the closest homologue in a particular species divided by the BlastP score of the protein against itself. CS values (represented by colors) can range from 0 when no homolog or ortholog is detected in another species, to 1 when the closest homolog exhibits 100% amino acid identity. The phylogenetic relationships shown to the left of the figures are based on orthologous housekeeping genes present in all species. Note that branch lengths do not represent phylogenetic distances.

The majority of genes directly regulated by PhoP in *Y. pestis* have no BLAST matches in *S. enterica* and vice versa (Figure 2 and Table S4). To determine whether the absence of homologs was particular to the *Yersinia*-*Salmonella* pairwise comparison, we searched the genomes of eight additional members of the family *Enterobacteriaceae* for the presence of sequences homologous to the identified PhoP-regulated protein-coding genes of *Yersinia* and *Salmonella*. This analysis revealed that over half of the gene products directly controlled by PhoP in *Yersinia* lack homologs in at least three other species (absence of homologous sequences is represented by white squares in Figure 2A). This finding indicates that these genes have been gained and/or lost during the evolution of the family *Enterobacteriaceae*. Analysis of the *S. enterica* PhoP regulon revealed a similar pattern as nearly half of the protein-coding genes directly regulated by PhoP had no homologs in most of the other enteric species (white area in Figure 2B). This is in parallel to the overall differences in gene content that exist between the phylogenetically most distant species in the analyzed group, *Escherichia coli* and *Y. pestis*, which share only ∼50% of their genes [19]. Consistent with the conclusions from a purely computational comparison of the *E. coli* and *S. enterica* PhoP regulons [27], our data indicate that orthologous PhoP proteins promote expression of largely distinct gene sets in individual enteric species.

While the biochemical function of the species-specific PhoP-activated gene products remains largely unknown, several of the *Salmonella*-specific targets have been implicated in survival within host cells, such as *mig-14* [28], or in resistance to host antimicrobial products, such as *ugtL* [29]. The majority of PhoP-activated *Yersinia*-specific targets encode uncharacterized proteins.

### PhoP-Regulated Protein-Coding Genes Exhibit Different Levels of Sequence Conservation in Enteric Bacteria

We determined the degree of sequence identity for each pair of homologs by calculating their conservation scores (CS) [30],[31], which represent the BlastP score of the closest homologue in a particular species divided by the BlastP score of the protein against itself. CS values range from 0 when no homolog or ortholog is detected in another species, to 1 when the closest homolog exhibits 100% amino acid identity. This analysis revealed that only three gene products directly controlled by the PhoP protein both in *Yersinia* and *Salmonella* – PhoP, PhoQ, and SlyB – are relatively well conserved in the analyzed enteric species (red squares in Figure 2A and 2B). By contrast, the well-conserved *psiE*, *ompX* and *rstA* genes are regulated by PhoP only in one of the two species.

A subset of the genes regulated directly by PhoP in either *Yersinia* or *Salmonella* encodes products poorly conserved in other enterics (blue squares in Figures 2A and 2B). These genes are unlikely to be true orthologs because the level of amino acid identity between the corresponding products is considerable lower than the median ∼72% identity that is found between *E. coli* and *Y. pestis* orthologs [19]) (blue in Figure 2 represents roughly 40% amino acid identity). For instance, PhoP promotes transcription of the *Salmonella pagP* gene and the *Yersinia y2563* gene by binding to their respective promoter regions (Figure 2). Even though the *pagP* and *y2563* genes are recovered as the best reciprocal hits in a BLAST search, their gene products are only 42% identical and do not seem to carry out the same biochemical function because the PagP-mediated palmitoylation of the lipid A established in *S. enterica* [32] has not been detected in *Y. pestis* [33]. Therefore, even when homologous genes are regulated in a like manner, the activities of their gene products may not be retained across species.

### Differences in the PhoP regulons of *Y. pestis* and *S. enterica* Resulting from Transcriptional Rewiring

The gains and/or losses of interactions between orthologous regulatory proteins and orthologous target genes (*i.e.*, transcriptional rewiring) account for the majority of differences in the targets of regulation among related eukaryotic species [3]–[5]. Rewiring events have not been considered in previous analyses of bacterial regulons because the purely computational comparisons reported to date have focused on gene content, and thus, they assumed that if a regulatory protein controls a particular target in one species, such regulatory relationship will be conserved in another species [34]–[36]. By combining ChIP-chip and expression data obtained with full genome tiling arrays, we could experimentally determine the contribution that rewiring events make to the composition of the PhoP regulons.

We established that the PhoP protein controls transcription of both the outer membrane-encoding gene *ompX* and the regulatory gene *rstA* in *Salmonella* but not in *Yersinia*. This may result in significant phenotypic differences between these two species because the *rstA* gene encodes a regulatory protein that modulates the levels of the alternative sigma factor RpoS [37] and of a Fur-repressed iron transporter [38] in *Salmonella*. Conversely, the phosphate-starvation inducible gene *psiE*, the putative aminidase encoding gene *y1877* (*ybjR*), and the putative inner membrane protein coding gene *y2124* (*STM3036*) are regulated by PhoP in *Yersinia* but not in *Salmonella*. Considering that ∼16 transcripts directly regulated by PhoP have homologous sequences in *Y. pestis* and *S. enterica*, this means that ∼30% of them have undergone transcriptional rewiring events. To our knowledge, this is the first report on the prevalence of transcriptional rewiring in a bacterial regulon based on experimental evidence.

In addition to changes in regulon membership, rewiring events can result in novel interactions between orthologous regulatory proteins and orthologous target genes that are qualitatively similar (*i.e.*, a target gene is turned on in response to the same signal) [39] but generate quantitatively different outputs. We determined that the *Yersinia* PhoP protein governs transcription of the *pbgP* (*y1917*) operon and the *ugd* (*y2147*) gene directly, by binding to their respective promoters (Table S3, see also [23]), but that it does so indirectly in *Salmonella* (*i.e.*, by activating a different regulatory protein) [40]–[42]. Though the direct and indirect pathways are qualitatively similar in the two species (*i.e.*, low Mg^2+^ promotes transcription of the polymyxin B-resistance conferring *pbgP* and *ugd* genes), the indirect pathway operating in *Salmonella* exhibits signal amplification and expression persistence relative to the direct pathway present in *Yersinia* [43].

### The Virulence Gene *mgtC* Was Independently Embedded in the PhoP Regulons of *Y. pestis* and *S. enterica*

The PhoP-activated *mgtC* gene encodes an inner membrane protein necessary for virulence in mice, survival within macrophages and growth in low Mg^2+^ in *Salmonella* [44], and for the survival of *Y. pestis* inside macrophages [10]. However, the low level of amino acid identity between the two MgtC proteins, the sporadic phylogenetic distribution of the *mgtC* gene within the family *Enterobacteriaceae* [44] as well as the presence of *mgtC* homologs in more distant bacterial species such as *Burkholderia cenocepacia* [45] raises the possibility that the *Salmonella* and *Yersinia mgtC* genes may be xenologs rather than orthologous (*i.e.*, acquired separately by *Salmonella* and *Yersinia* in independent horizontal-gene transfer events) and likely incorporated into the two PhoP regulons independently. This could reflect that the *mgtC* gene enables survival within mammalian cells and in Mg^2+^-limiting environments, which are conditions that activate the PhoP/PhoQ system [6].

### Autoregulation of the PhoP/PhoQ System Has Been Maintained in *Y. pestis* and *S. enterica* Despite Modifications in the *cis*-Regulatory Region of Its *phoP* Locus

**Figure 3:**
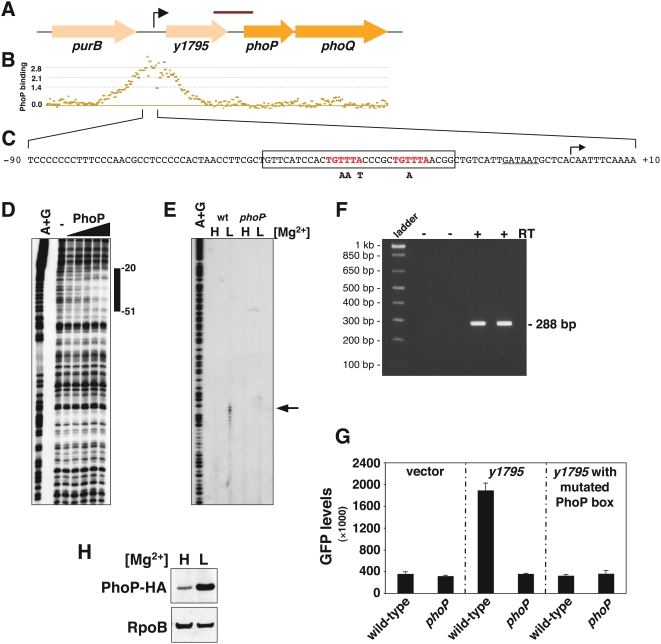
The *phoP* and *phoQ* genes are part of a PhoP-activated three-gene operon in *Y. pestis*. A) Diagram depicting the genomic context of the *phoPQ* locus in *Y. pestis*. The bent arrow shows the PhoP-activated transcription start site determined in (E). The red bar indicates the 288 nt transcript identified in (F). Large arrows represent protein-coding sequences. B) *In vivo* PhoP binding profile to the genomic region shown in (A) as determined by ChIP-chip. C) DNA sequence of the *y1795* promoter region. The transcription start site determined in (E) is indicated by the bent arrow; the PhoP binding site is in red; the putative −10 sequence is underlined, and the region protected by PhoP is boxed. Nucleotide substitutions introduced in the PhoP binding site in (G) are indicated below the box. D) DNase I footprinting analysis of the promoter region of the *y1795* gene with increasing amounts (0, 2.5, 5, 10 and 20 pmol) of *Y. pestis* PhoP protein. The bar indicates the protected region. E) Primer extension analysis of the *y1795* gene carried out with RNA samples prepared from wild-type (EG19221) and *phoP* mutant (EG14737) strains following growth under high (H) or low (L) Mg^2+^ concentrations. The arrow indicates the mapped transcription start site. F) Reverse-transcription-PCR analysis of RNA samples prepared from *Y. pestis* wild-type cells. Reverse-transcription was carried out with (+) or without (−) reverse-transcriptase (RT) and the products of these reactions used as template to PCR amplify the 288 nt fragment shown in (A). G) GFP expression driven by *Y. pestis* strains harboring plasmids with transcriptional fusions between the *y1795* promoter containing either wild-type sequence or point mutations in the predicted PhoP box and a promoterless *gfp* gene. Fluorescence was measured in wild-type (KIM6) and *phoP* (EG14737) strain backgrounds following growth in defined medium supplemented with 50 µM MgCl_2_. Shown are the mean and standard deviation values of at least three independent experiments performed in triplicates. H) Western blot analysis of cell extracts prepared from *Y. pestis* cells harboring an epitope tagged *phoP* gene (EG19221). Cells were grown as described in (E). Anti-RpoB antibodies were used to detect the RpoB protein, which served as a loading control.

The *Salmonella* PhoP/PhoQ system is transcriptionally autoregulated in a positive fashion by the PhoP protein binding to a promoter located immediately upstream of the *phoP* coding region [46],[47]. We established that the *Yersinia phoP* and *phoQ* genes were turned on in low Mg^2+^ in a PhoP-dependent fashion (Figure 3), just like the *Salmonella phoP* and *phoQ* genes [6]. However, a PhoP box could not be identified immediately upstream of the *phoP* coding region in *Yersinia* and the PhoP protein did not bind to this region *in vivo* (Figure 3A and 3B). We determined that positive autoregulation of the *Yersinia phoP* and *phoQ* genes is mediated by a promoter located upstream of *y1795*, the gene located 5′ of *phoP* and predicted to be transcribed in the same direction as the *phoP* and *phoQ* genes (Figure 3A) because: First, the PhoP protein bound to this region *in vivo* (Figure 3B) and footprinted it *in vitro* (Figure 3C and 3D). Second, transcription initiated at this promoter was *phoP*-dependent (Figure 3E) and extended into the *phoP* gene as determined by reverse transcription-PCR (Figure 3F). Third, point mutations in the predicted PhoP box of the *y1795* promoter abolished PhoP-dependent expression from this promoter (Figure 3G). And fourth, Western blot analysis of cell extracts prepared from a *Yersinia* strain encoding a PhoP-HA protein from its normal chromosomal location and probed with anti-HA antibodies demonstrated higher levels of PhoP-HA protein following growth under inducing (*i.e.*, low) than in repressing (*i.e.*, high) Mg^2+^ concentrations (Figure 3H).

The incorporation of the *y1795* gene into *Yersinia* appears to have occurred after the lineage that gave rise to this genus split from the one originating *Serratia* because *y1795* orthologs have not been found outside *Yersinia* spp. (see phylogeny in Figure 2). The *y1795* gene is predicted to encode a 207 amino acid outer membrane protein and/or lipoprotein without significant similarity to proteins of known function. That the *y1795* gene is adjacent to the *phoP* gene and co-transcribed with *phoP* and *phoQ* suggests that it may affect the levels and/or activities of the *Yersinia* PhoP and PhoQ proteins. While this possibility cannot be presently ruled out, chromosomal replacement of the *S. enterica phoP* and *phoQ* genes with the *Y. pestis* orthologs (without *y1795*) resulted in a strain that retained the normal regulation of the PhoP-activated *mgtA* gene [48]. Moreover, open reading frames harboring functions unrelated to those of the two-component system proteins sometimes precede two-component system genes. For instance, the *phoPQ* genes of *Pseudomonas aeruginosa* are preceded by and form an operon with *oprH*, which encodes an outer membrane protein [49]. Likewise, the genes for the PmrA/PmrB two-component system of *S. enterica* are part of a three-gene operon headed by the *pmrC* gene [50],[51], which encodes an inner membrane protein implicated in the modification of the lipopolysaccharide [52].

### Conserved PhoP-Regulated Targets Mediate Mg^2+^ Homeostasis

Mg^2+^ is the most abundant divalent cation in biological systems [53]. It is essential in the cytosol for ATP-mediated reactions and as a stabilizer of ribosomes [54], and in membranes where it binds to negatively charged molecules, such as the phosphates in the lipopolysaccharide [55]. Consistent with low Mg^2+^ being a signal that activates the PhoP/PhoQ system [6], several of the genes regulated by PhoP both in *Yersinia* and *Salmonella* and also present in the vast majority of the analyzed enteric species (Figure 2) encode proteins mediating the adaptation to low Mg^2+^.

On the one hand, the PhoP protein directly promotes transcription of the *Salmonella mgtA* and *mgtB* genes [26], encoding two of the three known Mg^2+^ transporters of *S. enterica* and exhibiting 50% identity to each other. PhoP directly regulates transcription of the *mgtCB* operon in *Y. pestis* (Figure S2), which lacks an *mgtA* gene. (The *mgtCB* operon also encodes the inner membrane protein MgtC discussed above.) Certain enteric species, such as *E. coli* and *Citrobacter koseri*, lack *mgtCB* but harbor *mgtA* (Figure 2B); other species such as *Serratia proteamaculans* and *S. enterica* harbor both *mgtA* and *mgtCB* whereas *Sodalis glossinidius* lacks homologs of all three genes. We identified putative PhoP boxes upstream of the coding regions of most of these Mg^2+^ transporter genes suggesting that the PhoP protein directly regulates their expression.

On the other hand, the proteins encoded by the PhoP-activated *pbgP* operon and the *ugd* gene are responsible for covalently modifying the lipid A phosphates in the lipopolysaccharide with 4-amino-4-deoxy-L-arabinose at sites normally neutralized by Mg^2+^, whereas the *pagP* gene product catalyzes the incorporation of a palmitate chain into lipid A [56]. These two modifications confer resistance to different antimicrobial peptides [57]. Except for *Citrobacter koseri*, all examined enteric species harbor *pbgP* and *pagP* homologs (Figure 2); yet, as discussed above, homologs may not be functionally equivalent.

Our analysis indicates that the PhoP regulon includes products that function in Mg^2+^ homeostasis, consistent with Mg^2+^ being the signal that regulates the PhoP/PhoQ system [6]. These products need not be highly conserved as long as they fulfill a required function. For example, *E. coli* and *Yersinia* harbor only one PhoP-activated Mg^2+^ transporter each, MgtA and MgtB, respectively, which are only 51% identical at the amino acid level, which is much lower than the 72% median identity that exists between *E. coli* and *Yersinia* proteins [19].

### Conserved Feedback Loops Control the Levels and the Activity of the PhoP/PhoQ System

**Figure 4:**
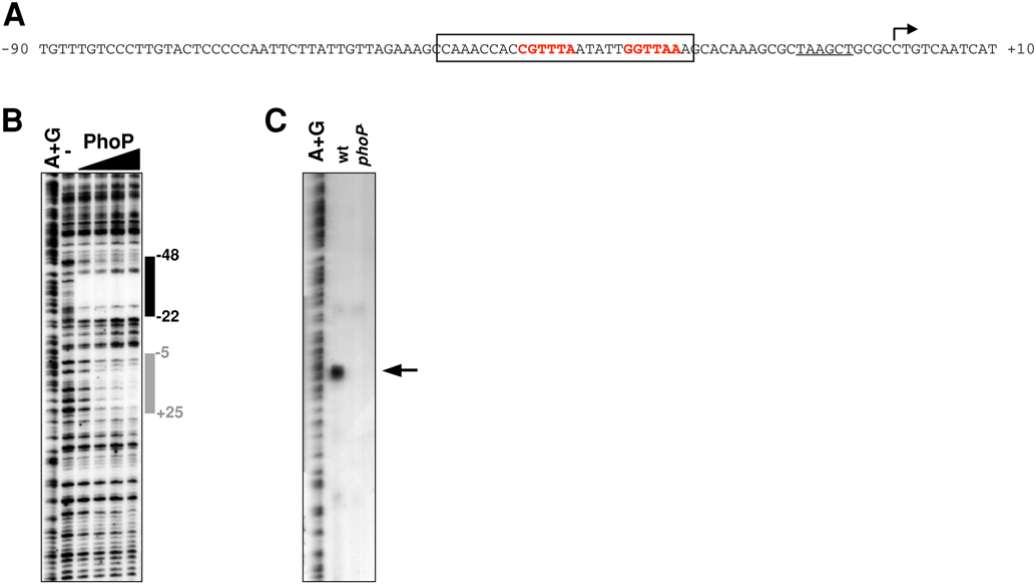
PhoP promotes transcription of the *Y. pestis slyB* gene. A) DNA sequence of the *Y. pestis slyB* promoter region. The transcription start site is indicated by the bent arrow; the PhoP binding site is in red; the putative −10 sequence is underlined, and the region protected by PhoP in (B) is boxed. B) DNase I footprinting analysis of the promoter region of the *Y. pestis slyB* gene with increasing amounts of *Y. pestis* PhoP protein (0, 5, 15, 30 and 60 pmol). The bars indicate protected regions. C) Primer extension analysis of the *Y. pestis slyB* gene carried out with RNA samples prepared from wild-type (EG19221) or *phoP* mutant (EG14737) strains following growth in low Mg^2+^. The arrow indicates the mapped transcription start site.

Only two loci are directly regulated by PhoP both in *Salmonella* and in *Yersinia* and present in all ten examined enteric species (Figure 2): *phoP/phoQ*, encoding the PhoP/PhoQ two-component regulatory system, and *slyB* (Figure 4), encoding an outer membrane lipoprotein with similarity to a lipoprotein implicated in membrane integrity in *Burkholderia* spp. [58]. Both *phoPQ* and *slyB* seem to be regulated by PhoP across the *Enterobacteriaceae* family because putative PhoP binding sites could be identified in their promoter regions in all analyzed enteric species (Figures S3 and S4). And in *E. coli*, direct PhoP regulation of *phoPQ* and *slyB* has been demonstrated experimentally [59].

Mutants lacking a functional *phoP* gene are defective for growth in low Mg^2+^ [6], consistent with PhoP's role in governing the adaptation to low Mg^2+^ environments. We determined that a *Salmonella* mutant deleted for the PhoP box in the *phoP* promoter exhibited an identical phenotype (Figure S5). This demonstrates that positive autoregulation of the *phoPQ* operon is required to generate sufficient amounts of active PhoP protein to promote expression of the PhoP-regulated gene products mediating growth in low Mg^2+^ [6],[26].

**Figure 5:**
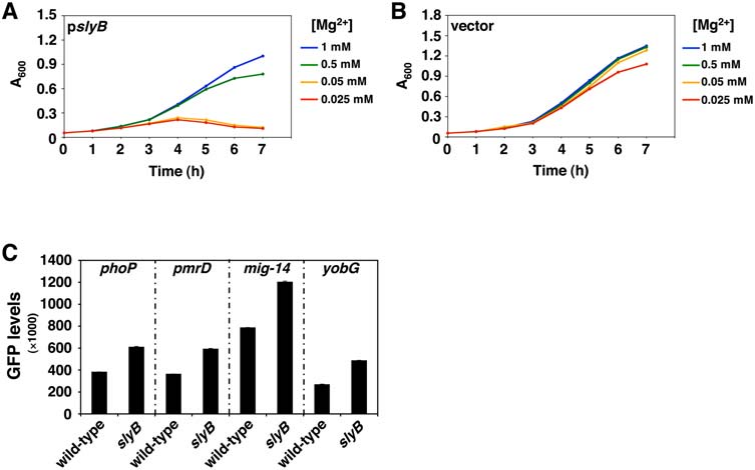
SlyB negatively regulates the activity of the PhoP/PhoQ regulatory system. A–B) Growth curves of wild-type *Salmonella* cells harboring plasmid pAHE-*slyB* over-expressing the *slyB* gene (A) or plasmid vector pAHE (B). Cells were grown in medium containing the Mg^2+^ concentrations indicated in the figure. 1 mM IPTG was added to all cultures to induce *slyB* expression. C) GFP expression driven by *Salmonella* strains harboring plasmids with transcriptional fusions between the promoters for the PhoP-activated genes *phoP*, *pmrD*, *mig-14*, and *yobG*, and a promoterless *gfp* gene. Fluorescence was measured in wild-type (14028 s) and *slyB* (DS292) strain backgrounds following growth in N-minimal medium supplemented with 50 µM MgCl_2_. Shown are the mean and standard deviation values of at least three independent experiments performed in triplicates.

To explore the *slyB* function, we examined the behavior of strains lacking or overexpressing the *slyB* gene and experiencing low Mg^2+^, which is a condition in which *Salmonella* requires a functional *phoP* gene [6],[26]. Whereas a *slyB*-deleted strain grew like wild-type *Salmonella*, the strain overexpressing *slyB* was defective for growth, especially in low Mg^2+^ (Figure 5A–B). To test whether the growth defect of the *slyB*-overexpressing strain was due to inhibition of PhoP protein activity, we examined transcription of four different PhoP-dependent promoters. We measured GFP expression levels in strains harboring transcriptional fusions to a promoterless *gfp* gene driven by either of four different PhoP-activated promoters in wild-type and *slyB Salmonella* strains grown under inducing conditions for the PhoP/PhoQ system. All promoters were expressed to higher levels in the *slyB* mutant compared to the isogenic wild-type strain (Figure 5C). The *slyB* gene was responsible for this phenotype because the production of the SlyB protein from a plasmid restored the wild-type levels of expression of the PhoP-activated *ugtL* gene to a *slyB* mutant strain harboring a chromosomal *lacZYA* transcriptional fusion to *ugtL* (Figure S6). Together, these results indicate that *slyB* negatively regulates the activity of the PhoP protein. This effect appears to be specific to *slyB* because transcription of PhoP-dependent targets was not altered when eight other PhoP-activated genes were mutated (data not shown).

The ability of the PhoP/PhoQ system to modulate its own activity through positive and negative feedback loops has been conserved over hundreds of millions of years. The key role that these regulatory loops play in the proper functioning of the PhoP/PhoQ system is underscored by the fact that altering the activity of this system by either overexpressing the negative regulator *slyB* or abolishing *phoPQ* positive autoregulation render *S. enterica* unable to grow in low Mg^2+^ (Figures 5A and S5). The use of regulatory feedbacks to control the output of two-component systems is not exclusive to PhoP/PhoQ because other two-component regulatory systems positively regulate their own expression [60] and because the CseB/CseC two-component system of *Streptomyces coelicolor* controls the expression of a lipoprotein that negatively regulates CseB/CseC activity [61]. (Note that even though the activity of the two-component systems CpxR/CpxA and RcsB/RcsC/RcsD is affected by the lipoproteins NlpE and RcsF, respectively, neither *nlpE* nor *rcsF* are controlled by the regulatory systems that they affect [62],[63].)

### Conclusions

The growing number of available bacterial genome sequences has revealed the existence of large extents of genomic variability even among closely related bacterial species. Despite these differences, closely related organisms typically rely on orthologous regulatory proteins to respond to a given stimulus. Here, we have explored how the content of bacterial regulons is shaped in an environment of widespread gains and losses of genes.

#### A multi-pronged approach to define regulons

Experimental studies aimed at uncovering regulons genome-wide have typically relied on only one of two approaches: expression microarray analysis or ChIP-chip. However, transcript analyses do not distinguish between direct and indirect targets and many *in vivo* binding sites for a regulatory protein do not direct transcription [64]. By utilizing *in vivo* binding data of a regulatory protein, genome-wide expression data of wild-type and mutant strains, and *in silico* analyses to define regulon membership, we could obtain an accurate estimate of rewiring in a bacterial regulon and avoid overestimating rewiring prevalence, which results when ChIP-chip data is solely considered (*e.g.*, see [3]–[5]).

#### Roles of core and variable members of bacterial regulons

Four aspects have shaped the PhoP regulon during the evolution of enteric bacteria: First, the acquisition and the subsequent loss of target genes; second, transcriptional rewiring of shared genes; third, differences in sequence conservation of the various targets of regulation; and fourth, retention of a small number of target genes. The latter defines a core regulon that controls the level of active PhoP protein and mediates the adaptation to low Mg^2+^, a condition activating the PhoP/PhoQ system [6]. By contrast, the members of the variable regulon aid bacterial proliferation in the particular niches preferred by individual species.

The notion that bacterial regulons consist of core and variable portions, as demonstrated in this study, is further supported by recent (largely computational) analyses of the RpoE regulon of enteric bacteria [65], where core regulon members determine the amount of the alternative sigma factor RpoE and maintain cell envelope integrity, the disruption of which increases the level of RpoE; and variable members of the regulon participate in virulence functions. Likewise, the targets of the alternative sigma factor PvdS of *Pseudomonas* spp. have been divided into a core group involved in the production of an iron chelator and an accessory group that is species-specific [66]. Finally, the DNA damage-activated LexA protein controls its own levels in most investigated organisms both by repressing its own transcription as well as that of the *recA* gene, which encodes a protein that promotes the autocatalytic cleavage (and resulting inactivation) of the LexA protein (reviewed in [67]). Conserved LexA regulon members participate in DNA repair and translesion synthesis and there is evidence that the broadly distributed LexA protein governs expression of a variable target set of genes [67], perhaps selected to respond in the specific habitats of individual species. Cumulatively, these findings suggest that the distinct roles played by core and variable portions of bacterial regulons are shared across structurally different regulatory proteins that operate by dissimilar mechanisms and respond to distinct signals.

## Materials and Methods

### Bacterial Strains, Plasmids, and Growth Conditions

Bacterial strains and plasmids used in this study are listed in Table S5. Primers are listed in Table S6. All *S. enterica* serovar Typhimurium strains were derived from wild-type strain 14028 s, and grown at 37°C in N-minimal medium [68] buffered in 50 mM Bis-Tris, pH 7.7, supplemented with 0.1% casamino acids, 38 mM glycerol and 50 µM or 10 mM MgCl_2_. *Y. pestis* strains were derived from wild-type strain KIM6 [69], and grown at the optimal growth temperature of 28°C in defined medium [70], pH 7.0, supplemented with 0.1% casamino acids, 10 mM (D)-glucosamine, and 50 µM or 10 mM MgSO_4_. *E. coli* strain DH5α was used as the host for the preparation of plasmid DNA. Ampicillin and kanamycin were used at 50 µg/ml and chloramphenicol at 20 µg/ml.

### RNA Isolation and Expression Microarray Analysis

After overnight culture in medium containing 10 mM MgSO_4_, *Y. pestis* cells were washed with Mg^2+^-free medium and grown to A_600_ ∼0.3 in 16 ml of medium containing 50 µM MgSO_4_ with vigorous shaking. 14 ml of cell culture were collected, mixed with RNAprotect™ Bacteria Reagent (Qiagen) and used to prepare total RNA using RNeasy® Mini Kit (Qiagen). RNA samples were treated with Turbo DNA-free DNase (Ambion) and re-purified with the RNeasy® Mini Kit. *Y. pestis* KIM tiling arrays were manufactured by NimbleGen Systems Inc (Madison). The array features are illustrated on Figure S1. RNA labeling, array hybridization and data extraction were carried out according to standard operating procedures by NimbleGen Systems Inc (Madison).

### Chromatin Immunoprecipitation - Microarray Analysis (ChIP-chip)

After overnight culture in medium containing 10 mM MgSO_4_, *Y. pestis* cells were washed with Mg^2+^-free medium and grown to A_600_ ∼0.3 in 22 ml of medium containing 50 µM MgSO_4_ with vigorous shaking. ChIP assays were carried out essentially as described [71]. DNA labeling (IP sample with Cy5; input DNA with Cy3), array hybridization, data extraction and analysis were carried out according to standard operating procedures by NimbleGen Systems Inc (Madison). Detailed information about these and other experimental protocols are provided in Text S1.

## Supporting Information

Figure S1 Tiling array design used for *Y. pestis* expression microarrays and ChIP-chip analyses. 50 nt oligos overlapping every 25 nt were designed to tile the entire *Y. pestis* KIM strain genome on both strands. Tiles on the Crick strand are offset 14 bp relative to those on the Watson strand.
(0.2 MB EPS)

Figure S2 PhoP regulates *mgtCB* transcription in *Y. pestis*. A) DNA sequence of the *Yersinia mgtCB* promoter region. The transcription start site identified in (B) is indicated by the bent arrow; the PhoP binding site is in red; the putative −10 sequence is underlined, and the region protected by PhoP (C) is boxed. B) Primer extension analysis of the *mgtCB* operon carried out with RNA samples prepared from wild-type (EG19221) or *phoP* mutant (EG14737) strains grown in defined medium containing 10 mM (H) or 50 µM (L) MgSO_4_ as described under Materials and Methods. The arrow indicates the mapped transcription start site, which is shown in (A). C) DNase I footprinting analysis of the promoter region of the *mgtCB* operon with a probe for the noncoding strand and increasing amounts of *Y. pestis* PhoP protein (0, 5, 10, 20 and 40 pmol). The bars indicate the protected regions. D) Occupancy of *mgtCB* promoter region by PhoP as determined by chromatin immunoprecipitation in a strain expressing PhoP-HA (EG19221) or in wild-type untagged cells (negative control) grown in defined medium containing 50 µM MgSO_4_ as described under Materials and Methods. Shown are the mean and S.D. values of three independent experiments.
(0.9 MB EPS)

Figure S3 Conservation of PhoP boxes in the *phoP* promoter across the *Enterobacteriaceae* family. Shown are the DNA sequences of the promoter regions of the *phoP* gene in ten enteric species whose genome sequences are available. Predicted PhoP boxes are in red, putative −10 sequences are underlined. The first eight amino acids of the *phoP* ORF are indicated below the nucleotide sequence. * The *Y. pestis* sequence corresponds to the *y1795* promoter and ORF.
(0.3 MB EPS)

Figure S4 Conservation of PhoP boxes in the *slyB* promoter across the *Enterobacteriaceae* family. Shown are the DNA sequences of the promoter regions of the *slyB* gene in nine enteric species whose genome sequences are available. Predicted PhoP boxes are in red, putative −10 sequences are underlined. The first eight amino acids of the *slyB* ORF are indicated below the nucleotide sequence.
(0.3 MB EPS)

Figure S5 Elimination of PhoP autoregulation impairs *Salmonella* growth in low Mg^2+^. Growth curves of *Salmonella* wild-type (EG13918 and p*_phoP_*::scar), Δ*phoP*/*phoQ* (EG15598) and a strain deleted for the PhoP box in the *phoP* promoter (EG14338). Cells were grown in N-minimal medium containing 10 µM MgCl_2_ with vigorous shaking at 37°C and A_600_ was determined every 60 min. The strain p*_phoP_*::scar harbors a “scar” sequence upstream of the PhoP box in the *phoP* promoter which does not influence PhoP expression [4].
(0.2 MB EPS)

Figure S6 SlyB negatively regulates the activity of the PhoP/PhoQ regulatory system. β-galactosidase activity (Miller units) expressed by *Salmonella* strains harboring a chromosomal *lac* transcriptional fusion to *ugtL* and either plasmid vector pAHE or plasmid pAHE-*slyB* expressing the *slyB* gene. Expression was investigated in wild-type (EG11250) and *slyB* (YS864) strain backgrounds following growth in N-minimal medium supplemented with 10 µM MgCl_2_. Shown are the mean and standard deviation values of at least three independent experiments performed in duplicates.
(0.2 MB EPS)

Table S1 PhoP regulated genes.
(1.9 MB XLS)

Table S2 Transcription units and ORFs whose expression is regulated by PhoP in *Y. pestis* based on tiling microarray data.
(0.08 MB DOC)

Table S3 Promoters/Transcripts directly activated by PhoP in *Y. pestis*. Summary of ChIP-chip data (3 biological replicates) for PhoP-bound regions (peaks) located nearby transcripts whose expression was *phoP*-activated.
(0.09 MB DOC)

Table S4 CS is the BlastP score of the closest homologue in a particular species divided by the BlastP score of the protein against itself. This can result in slightly different CS scores when *Salmonella* or *Yersinia* proteins are compared because the value in the denominator corresponds to the BlastP score of a protein against itself and *Salmonella* and *Yersinia* proteins are rarely identical. In other words, when scoring homologs of the *Salmonella* SlyB protein, the BlastP score of the *Salmonella* SlyB protein against itself will be in the denominator, whereas when scoring homologs of the *Yersinia* SlyB protein, the BlastP score of the *Yersinia* SlyB protein against itself will be in the denominator.
(0.06 MB DOC)

Table S5 Bacterial strains and plasmids used in this study.
(0.2 MB DOC)

Table S6 Primers used in this study.
(0.06 MB DOC)

Text S1 Supplementary text.
(0.07 MB DOC)
