# Supplementary material for: Evolution of a Bacterial Regulon Controlling Virulence and Mg2+ Homeostasis
Source: PLoS Genet. 2009 Mar 20;5(3):e1000428. doi: 10.1371/journal.pgen.1000428 (PMC2650801; doi:10.1371/journal.pgen.1000428)
Supplement: Table S2 — Transcription units and ORFs whose expression is regulated by PhoP in Y. pestis based on tiling microarray data. (0.08 MB DOC) [file pgen.1000428.s008.doc]

**Table S2.** Transcription units and ORFs whose expression is regulated by PhoP in *Y. pestis* based on tiling microarray data.

| **TRANSCRIPTION UNITS ACTIVATED BY PHOP** | | |
| --- | --- | --- |
| **Transcript name** | **ORFs included in the transcript** | **Direct regulation by PhoP?** |
| *psiE* | *psiE* | yes |
| *y1055* | *y1054, y1055* | no |
| *y0239* | *y0239* | no |
| *cstA* | *cstA* | no |
| *y0447* | *y0447* | yes |
| *y0838* | *y0838, y0839, y0840* | no |
| *nqrB* | *nqrB* | no |
| *y1306* | *y1306* | no |
| *y1579* | *y1579, y1580* | no |
| *fabB* | *fabB* | no |
| *y1795* | *y1795, phoP, phoQ, y1792* | yes |
| *y1803* | *y1803* | no |
| *mgtC* | *mgtC, mgtB* | yes |
| *y1877* | *y1877* | yes |
| *y1917 (pbgP)* | *y1917, y1718, y1919, y1720, y1921, y1722, y1923* | yes |
| *slyB* | *slyB* | yes |
| *y2124* | *y2124* | yes |
| *y2147 (ugd)* | *y2147* | yes |
| *crcA (pagP)* | *crcA* | yes |
| *y2608* | *y2608* | yes |
| *y2814* | *y2814, y2813* | no |
| *y2816* | *y2816* | yes |
| *y2858* | *y2858, y2859* | no |
| *y2868* | *y2868* | no |
| *y3093* | *y3093* | no |
| *y3284* | *y3284* | yes |
| *y3554* | *y3554, y3553* | no |
| *up_y3808* | *upstream of y3808 (no ORF)* | yes |
| *y3948* | *y3948* | yes |
| *y4116* | *y4116* | no |
| *y4126* | *y4126, y4125, gltJ, y4123* | yes |
| **TRANSCRIPTION UNITS REPRESSED BY PHOP** | | |
| *y0181* | *y0181* | no |
| *y0566* | *y0566* | no |
| *y0666* | *y0666* | no |
| *y0961* | *y0961* | no |
| *y0962* | *y0962* | no |
| *tcaA1* | *tcaA1* | no |
| *vacJ* | *vacJ* | no |
| *y1048* | *y1048* | no |
| *y1730* | *y1730, y1729, y1728* | no |
| *y2943* | *y2943, y2942* | no |
| *y2948* | *y2948, y2947, y2946, y2945* | no |
| *ibpA* | *ibpA, ibpB* | yes |
| *ompC* | *ompC* | no |
| *ftsW* | *ftsW* | yes |
