# Supplementary material for: Evolution of a Bacterial Regulon Controlling Virulence and Mg2+ Homeostasis
Source: PLoS Genet. 2009 Mar 20;5(3):e1000428. doi: 10.1371/journal.pgen.1000428 (PMC2650801; doi:10.1371/journal.pgen.1000428)
Supplement: Table S3 — Promoters/Transcripts directly activated by PhoP in Y. pestis. Summary of ChIP-chip data (3 biological replicates) for PhoP-bound regions (peaks) located nearby transcripts whose expression was phoP-activated. (0.09 MB DOC) [file pgen.1000428.s009.doc]

**Table S3.** Promoters/Transcripts directly activated by PhoP in *Y. pestis*.

Summary of ChIP-chip data (3 biological replicates) for PhoP-bound regions (peaks) located nearby transcripts whose expression was *phoP*-activated.

| **gene_ID** | **array #** | **Chromosome coordinates (*Y. pestis* KIM)** | | **score** | **FDR** |
| --- | --- | --- | --- | --- | --- |
|  |  | **peak start** | **peak end** |  |  |
| *psiE* | 1 | 39589 | 40106 | 1.94 | 0.00E+00 |
|  | 2 | 39654 | 40015 | 1.92 | 0.00E+00 |
|  | 3 | 39537 | 40080 | 1.78 | 0.00E+00 |
| *y0447* | 1 | 498828 | 499189 | 1.34 | 4.39E-02 |
|  | 2 | 498841 | 499046 | 1.15 | 1.79E-02 |
|  | 3 | 498867 | 499254 | 1.07 | 7.80E-02 |
| *y1795* | 1 | 1976339 | 1977143 | 1.94 | 0.00E+00 |
|  | 2 | 1976495 | 1977065 | 1.92 | 0.00E+00 |
|  | 3 | 1976352 | 1977156 | 1.78 | 0.00E+00 |
| *mgtC* | 1 | 2002002 | 2002467 | 1.94 | 0.00E+00 |
|  | 2 | 2002080 | 2002467 | 1.83 | 0.00E+00 |
|  | 3 | 2002028 | 2002467 | 1.62 | 0.00E+00 |
| *y1877* | 1 | 2073872 | 2074181 | 1.94 | 0.00E+00 |
|  | 2 | 2073898 | 2074181 | 1.58 | 1.25E-03 |
|  | 3 | 2073859 | 2074194 | 1.46 | 1.30E-02 |
| *y1917 (pbgP)* | 1 | 2109757 | 2110638 | 1.94 | 0.00E+00 |
|  | 2 | 2109783 | 2110638 | 1.92 | 0.00E+00 |
|  | 3 | 2109627 | 2110664 | 1.78 | 0.00E+00 |
| *slyB* | 1 | 2162967 | 2163198 | 1.94 | 0.00E+00 |
|  | 2 | 2162967 | 2163172 | 1.36 | 4.14E-03 |
|  | 3 | 2162941 | 2163198 | 1.56 | 0.00E+00 |
| *y2124* | 1 | 2346169 | 2346452 | 1.89 | 3.29E-03 |
|  | 2 | 2346247 | 2346556 | 1.45 | 1.53E-03 |
|  | 3 | 2346247 | 2346426 | 1.52 | 9.67E-03 |
| *y2147 (ugd)* | 1 | 2373831 | 2374152 | 1.94 | 0.00E+00 |
|  | 2 | 2373818 | 2374191 | 1.58 | 1.25E-03 |
|  | 3 | 2373831 | 2374178 | 1.7 | 6.86E-03 |
| *y2563* (*pagP*) | 1 | 2826265 | 2826496 | 1.48 | 2.57E-02 |
|  | 2 | 2826213 | 2826496 | 1.13 | 1.86E-02 |
|  | 3 | 2826278 | 2826457 | 1.13 | 6.56E-02 |
| *y2608* | 1 | 2873838 | 2874303 | 1.94 | 0.00E+00 |
|  | 2 | 2873890 | 2874225 | 1.92 | 0.00E+00 |
|  | 3 | 2873799 | 2874290 | 1.78 | 0.00E+00 |
| *y2816* | 1 | 3118816 | 3118943 | 0.99 | 1.31E-01 |
| *y3284* | 1 | 3614807 | 3615272 | 1.94 | 0.00E+00 |
|  | 2 | 3614859 | 3615246 | 1.77 | 0.00E+00 |
|  | 3 | 3614911 | 3615324 | 1.78 | 0.00E+00 |
| *up_y3808* | 1 | 4231037 | 4231393 | 1.64 | 1.07E-02 |
|  | 2 | 4231154 | 4231380 | 1.26 | 1.00E-02 |
|  | 3 | 4231076 | 4231380 | 1.34 | 2.43E-02 |
| *y3948* | 1 | 4403679 | 4403910 | 1.85 | 6.58E-03 |
|  | 2 | 4403718 | 4403871 | 1.54 | 9.93E-04 |
|  | 3 | 4403731 | 4403884 | 1.5 | 1.05E-02 |
| *y4126* | 1 | 4580439 | 4580982 | 1.94 | 0.00E+00 |
|  | 2 | 4580673 | 4580930 | 1.62 | 1.55E-03 |
|  | 3 | 4580673 | 4580956 | 1.78 | 0.00E+00 |
