# Supplementary material for: Evolution of a Bacterial Regulon Controlling Virulence and Mg2+ Homeostasis
Source: PLoS Genet. 2009 Mar 20;5(3):e1000428. doi: 10.1371/journal.pgen.1000428 (PMC2650801; doi:10.1371/journal.pgen.1000428)
Supplement: Table S4 — CS is the BlastP score of the closest homologue in a particular species divided by the BlastP score of the protein against itself. This can result in slightly different CS scores when Salmonella or Yersinia proteins are compared because the value in the denominator corresponds to the BlastP score of a protein against itself and Salmonella and Yersinia proteins are rarely identical. In other words, when scoring homologs of the Salmonella SlyB protein, the BlastP score of the Salmonella SlyB protein against itself will be in the denominator, whereas when scoring homologs of the Yersinia SlyB protein, the BlastP score of the Yersinia SlyB protein against itself will be in the denominator. (0.06 MB DOC) [file pgen.1000428.s010.doc]

**Table S4**. Conservation scores (CS) of proteins encoded by genes directly activated by PhoP in *S. enterica* and *Y. pestis*.

| *Salmonella* PhoP-regulated gene product | CS of *Salmonella* homologs in *Yersinia* |  | *Yersinia* PhoP-regulated gene product | CS of *Yersinia* homologs in *Salmonella* |
| --- | --- | --- | --- | --- |
|  |  |  |  |  |
| PhoP | 0.819 |  | PhoP | 0.819 |
| SlyB | 0.793 |  | SlyB | 0.790 |
| RstA | 0.715 |  | MgtC | 0.597 |
| MgtC | 0.604 |  | Y4126 | - |
| PagP | 0.445 |  | Y0447 | - |
| YbjX | 0.396 |  | Y2563 | 0.433 |
| PagK | - |  | Y1795 | - |
| UgtL | - |  | Y1917 | 0.699 |
| PagC | - |  | Y2147 | - |
| Mig-14 | - |  | Y2816 | 0.391 |
| PcgL | - |  | Y1877 | 0.440 |
| VirK | - |  | Y2124 | 0.455 |
| PipD | - |  | Y3284 | - |
| OmpX | 0.680 |  | Y2608 | - |
| PmrD | - |  | psiE | 0.667 |
| YrbL | - |  | Y3948 | - |
| YobG | - |  | MgtB | 0.734 |
| MgtA | - |  | PhoQ | 0.625 |
| IraP | - |  |  |  |
| PhoN | - |  |  |  |
| MgtB | 0.714 |  |  |  |
| PhoQ | 0.601 |  |  |  |
