# Supplementary material for: Evolution of a Bacterial Regulon Controlling Virulence and Mg2+ Homeostasis
Source: PLoS Genet. 2009 Mar 20;5(3):e1000428. doi: 10.1371/journal.pgen.1000428 (PMC2650801; doi:10.1371/journal.pgen.1000428)
Supplement: Table S5 — Bacterial strains and plasmids used in this study. (0.2 MB DOC) [file pgen.1000428.s011.doc]

**Table S5**. Bacterial strains and plasmids used in this study.

| Strain or plasmid | Description | Reference or source |
| --- | --- | --- |
| S. enterica |  |  |
| 14028s | Wild-type | ATCC |
| EG15598 | Δ*phoP/phoQ*::Cm^R^ | [1] |
| EG13918 | *phoP*-HA | [1] |
| DS292 | Δ*slyB*::Cm^R^ | This work |
| EG14338 | ΔPhoP box on phoP promoter phoP-HA | This work |
| EG11250 | ugtL::lac-Km^R^ | [15] |
| YS864 | ugtL::lac-Km^R^ ΔslyB | Y Shi and EA Groisman (unpublished) |
| Y. pestis |  |  |
| KIM6 | Pgm^-^ Pst^+^ Lcr^-^ Fra^+^ (pmT1, pCP1) | [16] |
| EG14737 | Δ*phoP*::Cm^R^ | [14] |
| EG19221 | *phoP*-HA | This work |
| E. coli |  |  |
| DH5α | F^-^ *sup*E44 Δ*lac*U169 (φ80 *lacZ*ΔM15) *hsd*R17 *rec*A1 *end*A1 *gyr*A96 *thi*-1 *rel*A1 | [17] |
| ER2566 | *fhuA2* [*lon*] *ompT lacZ*::T7 *gene1 gal sulA11* Δ(*mcrC-mrr*)*114*::IS*10* R(*mcr-73*::miniTn*10*-Tet^S^)2 R(*zgb-210*::Tn*10*-Tet^S^) *endA1* [*dcm*] | New England Biolabs |
| EG17025 | ER2566 Δ*phoPQ*::Km^R^ | [18] |
| Plasmids |  |  |
| pT7-7 | rep_PMB1_ Ap^R^ p_T7_ | [19] |
| pT7.7-PhoP*_Yersinia_*-His6 | rep_PMB1_ Ap^R^ p_T7_ *phoP*-His6 | This work |
| pAHE | rep_pACYC184_ *lacI^q^* Cm^R^ | This work |
| pAHE-*slyB* | rep_pACYC184_ *lacI^q^* Cm^R^ *slyB* | This work |
| pMS201 | rep_pSC101_ Km^R^ *gfp* | [20] |
| pMS201-p*_y1795_* | rep_pSC101_ Km^R^ p*_y1795_* *gfp* | This work |
| pMS201-p*_y1795-mut_* | rep_pSC101_ Km^R^ p*_y1795-mut_ gfp* | This work |
| pMS201-p*_phoP_* | rep_pSC101_ Km^R^ p*_phoP_* *gfp* | This work |
| pMS201-p*_pmrD_* | rep_pSC101_ Km^R^ p*_pmrD_ gfp* | This work |
| pMS201-p*_mig-14_* | rep_pSC101_ Km^R^ p*_mig-14_ gfp* | This work |
| pMS201-p*_yobG_* | rep_pSC101_ Km^R^ p*_yobG_ gfp* | This work |
| pKD3 | rep_R6Kγ_ Ap^R^ FRT Cm^R^ FRT | [3] |
| pKD46 | rep_pSC101_ts Ap^R^ p*_araBAD_* γ β exo | [3] |
| pCP20 | rep_pSC101_ts Ap^R^ Cm^R^ *cl*857 λP_R_ *flp* | [21] |
