# Supplementary material for: Evolution of a Bacterial Regulon Controlling Virulence and Mg2+ Homeostasis
Source: PLoS Genet. 2009 Mar 20;5(3):e1000428. doi: 10.1371/journal.pgen.1000428 (PMC2650801; doi:10.1371/journal.pgen.1000428)
Supplement: Table S6 — Primers used in this study. (0.06 MB DOC) [file pgen.1000428.s012.doc]

**Table S6**. Primers used in this study.

| Primer number | Sequence | Gene |
| --- | --- | --- |
| *Strain construction* | |  |
| 6320 | 5’-ATGCTGAAATAGCGTTTCGTGAAGAACAGGAGA TGGGTAACATATGAATATCCTCCTTAG-3’ | *slyB** |
| 6321 | 5’-GGGCATACGTGTGGCCATGTGACCACACGTAAA GCCTGGTTGTAGGCTGGAGCTGCTTCG-3’ | *slyB** |
| 2868 | 5’-TCACCCTCTTTTCTTCAGAAAGAGGGTGACTATT  TGTCTGCATATGAATATCCTCCTTAG-3’ | *phoP** |
| 2869 | 5’-AGTCTAGCGTTGATTATGGTGCTTTGGGGATAAA  CAGTTATGTAGGCTGGAGCTGCTTCG-3’ | *phoP** |
| Plasmid construction | |  |
| 6078 | 5’-CGGGATCCGAACAGGAGATGGGTAAATGATTA  AAC-3’ | *slyB** |
| 6079 | 5’-AACTGCAGCTGGTTTAGCGTGGAGAGACGGT  AAC-3’ | *slyB** |
| 7510 | 5'-CGGCATATGCGGGTTCTGGTTGTGGAAG-3' | *phoP* |
| 7517 | 5'-GCCAAGCTTAGTGGTGGTGGTGGTGGTGGTTGAC  GTCAAAACGATATCC-3' | *phoP* |
| 9107 | 5'-GCGCTCGAGGTAAAAGCTAACGCCATCTG-3' | *y1795* |
| 9104 | 5'-GCGGGATCCCCTGTGATTGTAACGTCCTTTTG-3' | *y1795* |
| *Site-directed mutagenesis* | |  |
| 9978 | 5'-CCTTCGCTGTTCATCCACTGAATTCCCGCTG  ATTAACGGCTGTCATTGATAA-3' | *y1795* |
| 9979 | 5'-TTATCAATGACAGCCGTTAATCAGCGGGAATT  CAGTGGATGAACAGCGAAGG-3' | *y1795* |
| *Reverse transcription-PCR* | |  |
| 9101 | 5'-ACGCGTTATCTTCCACAACC-3' | *y1795-phoP* |
| 9102 | 5'-GAGTCAGTGACCAAGCGATG-3' | *y1795-phoP* |
| Quantification of ChIP DNA by real-time PCR | |  |
| 9040 | 5'-GCACTAGCCAGAAAAAAATGAAACA-3' | *rpoD* |
| 9030 | 5'-AGTTGCCCCCACATTTATCG-3' | *rpoD* |
| 9034 | 5'-ATTATTCCTTCCCCCCCTTTC-3' | *y1795* |
| 9031 | 5'-AGTGGATGAACAGCGAAGGTTAG-3' | *y1795* |
| 9038 | 5’- TTT GGAAAATTACGATGGAGAAA-3’ | *mgtC* |
| 9041 | 5’- TTGACTTGTACTTTGCGTATCCA-3’ | *mgtC* |
| *Primer extension and DNase I footprinting* | |  |
| 8966 | 5'-CACCACCATGGTTGATGAAT-3' | *y1795* |
| 8967 | 5'-CAGGGCAAGGGATAATAGGG-3' | *y1795* |
| 9233 | 5'-ATCGCCATTGGAACAATCTT-3' | *slyB* |
| 9248 | 5'-CAAATCCGGCACTGTCAAAAG-3' | *slyB* |
| 8950 | 5'-CGTTATCGAGGTCTGTTGAATC-3' | *mgtC* |
| 8951 | 5'-CACCGAGAGGGTGCTGCCCGC-3' | *mgtC* |

*Indicates *Salmonella* sequence
