# Supplementary material for: Evolution of a Bacterial Regulon Controlling Virulence and Mg2+ Homeostasis
Source: PLoS Genet. 2009 Mar 20;5(3):e1000428. doi: 10.1371/journal.pgen.1000428 (PMC2650801; doi:10.1371/journal.pgen.1000428)
Supplement: Text S1 — Supplementary text. (0.07 MB DOC) [file pgen.1000428.s013.doc]

#### Text S1

**Materials and Methods**

### Construction of bacterial strains and plasmids

The *Y. pestis* strain harboring a chromosomally-encoded HA-tagged *phoP* gene was constructed as described [1, 2]. The structure of the modified region was verified by colony PCR [3] and DNA sequencing. The *Salmonella* strain deleted for the *slyB* gene was constructed using the one-step gene inactivation method [3]. The Cm^R^ cassette from plasmid pKD3 was amplified using primers 6320 and 6321 (primer sequences are described in Table S6) and the resulting PCR product integrated into the *slyB* locus in strain 14028s. The *Salmonella* strain in which the PhoP box in the chromosomal *phoP* promoter region of strain EG13918 is deleted was constructed as described [4] using primers 2868 and 2869.

Plasmid pT7.7-PhoP*_Yersinia_*-His6 was constructed by cloning a DNA fragment generated by PCR with primers 7510 and 7517 and genomic DNA from wild-type *Y. pestis* as template between the NdeI and HindIII sites of plasmid pT7.7.

To construct plasmid pAHE-*slyB* in which the SlyB protein is expressed from the p*_lac_* promoter, the *slyB* gene was amplified by PCR using primers 6078 and 6079 and chromosomal DNA from strain 14028s as template. This DNA fragment was cloned between the BamHI and PstI sites of pAHE.

The GFP reporter plasmids were constructed by cloning PCR-generated DNA fragments corresponding to the *Y. pestis y1795* and the *S. enterica* *phoP*, *mig-14*, *pmrD*, and *yobG* promoter regions between the XhoI and BamHI of plasmid pMS201. Mutagenesis of the plasmid harboring the *y1795* promoter region was carried out with primers 9978-9979 using the QuikChange Site-Directed Mutagenesis Kit (Stratagene) following the manufacturer’s instructions.

Unless specified otherwise, all PCRs were carried out with AccuPrime Taq DNA Polymerase High Fidelity (Invitrogen) and the correct sequence of the constructs was verified by DNA sequencing.

### Expression microarray analysis

The expression microarray experiments were conducted in triplicates. Systematic error [5] was treated by a the Moderated t-Test [6], which is similar to the Student’s t-Test in that it is used to compare the means of probe expression values for replicates for a given gene. The Student’s t-Test calculates variance from the data that is available for each gene, while the Moderated t-Test uses information from all of the selected probes to calculate variance. To correct for multiple tests (i.e., false positives within a large dataset) we used the Benjamini Hochberg method [7], which is not as conservative as the Bonferroni approach. This method aims to reduce the False Discovery Rate (FDR) and is used when the objective is to reduce the number of false positives and to increase the chances of identifying all the differentially expressed genes. In this method, the p-values are first sorted and ranked. The smallest value gets rank 1, the second rank 2, and the largest gets rank N. Then, each p-value is multiplied by N and divided by its assigned rank to give the adjusted p-values. In order to restrict the false discovery rate to 0.05, all the probes with adjusted p-values < 0.05 were selected.

We selected probes that exhibited differential expression through all six experiments. A list of all the probes that exhibited differential expression (>2-fold) in the microarrays is provided in Table S1. Overall, 1463, 1998, and 2319 probes were identified at 99%, 95%, and 90% of confidence, respectively. The differentially expressed transcripts were identified by collating the extracted probe locations with the *Y. pestis* KIM genome (NCBI NC_004088.1). After identifying the significant probe sets, we used them to calculate the expression significance (*i.e.*, fold change) of a complete gene and/or transcript. To do so, we performed a weighted sum of the individual fold changes *F* corresponding to the probe sets *j* overlapping the coding region of a gene *i* : . Then, we applied a two class unpaired test using SAM 3.05 [8, 9] with T-test and Delta = 25, estimated miss rates of 99.88% in average and FDR<0.05. These settings generated resulting genes/transcripts harboring fold changes >2.5, which are significantly higher than the fold changes of the random probe sets included in the array.

### Real-time PCR to determine transcript levels and reverse-transcription PCR

cDNA was synthesized using TaqMan (Applied Biosystems) and random hexamers following the manufacturer’s instructions. Quantification of transcripts was performed by real-time PCR using SYBR Green PCR Master Mix (Applied Biosystems) in an ABI 7000 Sequence Detection System (Applied Biosystems). Results were normalized to the levels of 16S ribosomal RNA. The amount of each PCR product was calculated from standard curves obtained from PCRs with the same primers and serially diluted DNA.

Primers 9101 and 9102 were used to amplify the fragment between the 3’ end of the *y1795* gene and the 5’ region of the *phoP* gene from cDNA prepared as described above.

### Chromatin immunoprecipitation - microarray analysis (ChIP-chip)

ChIP assays were carried out as described [1] with the following modifications: PhoP-HA-crosslinked DNA was immunoprecipitated with anti-HA H6908 antibodies (Sigma) and the latter captured with Protein G sepharose (GE Healthcare). After reversal of crosslinking, the immunoprecipitated (IP) and input DNA were purified using QIAquick columns (Qiagen) following manufacturer’s instructions. To generate enough material for hybridization, two rounds of genome amplification were carried out with the IP and input DNA samples as described [10] using the GenomePlex Complete Whole Genome Amplification Kit (Sigma). We performed three independent ChIP assays.

The ChIP microarray (ChIP-chip) data were analyzed as follows. Signal intensity data was extracted from the scanned images of each array using NimbleScan. A scaled log_2_-ratio of the co-hybridized input and IP samples was calculated for each tile on the array. The log_2_-ratio was computed and scaled to center the ratio data around zero. Scaling was performed by subtracting the bi-weight mean for the log_2_-ratio values for all tiles on the array from each log_2_-ratio value. Peaks were detected by searching for 4 or more tiles whose signals were above a cutoff value (ranging from 90% to 15% of a hypothetical maximum defined as the mean + 6 standard deviations) using a 500 bp sliding window. The ratio data was then randomized 20 times to evaluate the probability of false positives. Each peak was then assigned a false discovery rate (FDR) score based on the randomization. The analyzed data were visualized using NimbleGen SignalMap software.

Quantification of the IP and input DNA was performed by real-time PCR using SYBR Green PCR Master Mix (Applied Biosystems) in an ABI 7000 Sequence Detection System (Applied Biosystems). For amplification of the *rpoD*, *y1795,* and *mgtC* promoter regions, primers 9040 and 9030; 9034 and 9031; and 9038 and 9041 were used, respectively. The amount of each PCR product was calculated from standard curves obtained from PCRs with the same primers and serially diluted DNA. The level of protein binding to a particular promoter under each condition was calculated as follows:

promoter *x* IP / promoter *x* input

Relative protein bound to promoter *x* = ­

*rpoD* IP / *rpoD* input

The *rpoD* promoter neither binds to nor is regulated by the PhoP protein.

### Western blot analysis

Cells were grown as described above for RNA isolation, washed with TBS twice, resuspended in 300 μl TBS and opened by sonication. Whole-cell lysates were electrophoresed on 10% NuPAGE Bis-Tris gels (Invitrogen) with MES running buffer, transferred to nitrocellulose membranes and analyzed by immunoblotting with anti-HA (Sigma) or anti-RpoB (Neoclone) monoclonal antibodies. Blots were developed by using anti-mouse IgG horseradish peroxidase-linked antibodies (Amersham Biosciences) and the ECL detection system (Amersham Biosciences).

*Measurement of bacterial growth*

Growth of wild-type *Salmonella* strains harboring either the *slyB*-expressing plasmid or the plasmid vector was monitored as follows. Overnight cultures were grown in N-minimal medium containing 10 mM MgCl_2_, washed twice with Mg^2+^-free medium and diluted (1:50) in 150 ml flasks containing 15 ml of N-minimal medium supplemented with various concentrations of MgCl_2_ and 1 mM IPTG. Cells were grown with vigorous shaking at 37°C and the optical density (A_600_) of the cultures was measured every hour.

### GFP expression

After overnight culture in N-minimal medium containing 10 mM MgCl_2_, the *Salmonella* strains harboring GFP reporter plasmids were washed twice with Mg^2+^-free medium and inoculated (1:50 dilution) in a 96-well plate (Packard) containing 100 μl of N-minimal medium supplemented with 50 μM MgCl_2_. After overlaying the wells with 100 μl of mineral oil (Sigma) to prevent evaporation of medium, the plate was inserted into a Victor^3^ 1420 Multilabel counter (PerkinElmer) pre-warmed at 37°C. Fluorescence and cell density were monitored every ~6 min following plate shaking (1 min with 0.1 mm diameter), and this protocol was repeated for ~7 hours. The GFP values reported in Fig. 5C correspond to the fluorescence values after ~6h divided by the cell density at the same time. The cell cultures of wild type and mutant strains were similar in cell density during the entire growth period.

*β-galactosidase assays*

Cells were grown overnight in N-minimal medium containing 10 mM MgCl_2_, washed twice with Mg^2+^-free medium and inoculated (1:50 dilution) in N-minimal medium containing 10 μM MgCl_2_. Activity was determined as described elsewhere [11] after 4 h of growth at 37°C.

### Overproduction and purification of proteins

The *Yersinia* PhoP-His6 protein was overproduced in *E. coli* strain EG17025 harboring pT7-7-PhoP*_Yersinia_*-His6. Protein purification was performed as described [12] with the following modifications: after purification, the buffer of the eluate was exchanged with 10 mM Hepes (pH 8.0), 10% (v/v) glycerol and the protein was concentrated using an Amicon Ultra-15 column (MW 10,000; Millipore). The protein was stored at -80°C. Protein concentration was determined with a BCA protein assay (Pierce) using BSA as standard. Protein preparations were >99% pure as determined by SDS-PAGE followed by Coomassie blue staining.

### Primer extension and DNase I footprinting

RNA samples were prepared as described above for expression microarrays. Primer extension assays were performed as reported [13] with primers listed in Table S6.

DNase I footprinting with the *Y. pestis* PhoP-His6 protein was performed as reported [14]. The *y1795*, *slyB,* and *mgtC* promoter regions were PCR amplified with primers 8966 and 8967; 9233 and 9248; and 8950 and 8951, respectively, and genomic DNA of wild-type *Y. pestis* as template.

## References

1. Shin D, Groisman EA (2005) Signal-dependent binding of the response regulators PhoP and PmrA to their target promoters *in vivo*. J Biol Chem 280: 4089-4094.

2. Uzzau S, Figueroa-Bossi N, Rubino S, Bossi L (2001) Epitope tagging of chromosomal genes in *Salmonella*. Proc Natl Acad Sci USA 98: 15264-15269.

3. Datsenko KA, Wanner BL (2000) One-step inactivation of chromosomal genes in *Escherichia coli* K-12 using PCR products. Proc Natl Acad Sci USA 97: 6640-6645.

4. Shin D, Lee EJ, Huang H, Groisman EA (2006) A positive feedback loop promotes transcription surge that jump-starts *Salmonella* virulence circuit. Science 314: 1607-1609.

5. Nadon R, Shoemaker J (2002) Statistical issues with microarrays: processing and analysis. Trends Genet 18: 265-271.

6. Smyth GK (2004) Linear models and empirical Bayes methods for assessing differential expression in microarray experiments. Stat Appl Genet Mo B 3: 3.

7. Benjamini Y, Hochberg Y (1995) Controlling the False Discovery Rate. A Practical and Powerful Approach to Multiple Testing. J Roy Stat Soc B Met 57: 289-300.

8. Efron B, Tibshirani R (2002) Empirical Bayes methods and false discovery rates for microarrays. Genet Epidemiol 23: 70-86.

9. Efron B, Tibshirani R, Storey JD, Tusher V (2001) Empirical Bayes analysis of a microarray experiment. J Am Stat Assoc 96: 1151-1160.

10. O'Geen H, Nicolet CM, Blahnik K, Green R, Farnham PJ (2006) Comparison of sample preparation methods for ChIP-chip assays. Biotechniques 41: 577-580.

11. Miller JH (1972) Experiments in molecular genetics. Cold Spring Harbor, NY: Cold Spring Harbor Laboratory Press.

12. Wosten MMSM, Groisman EA (1999) Molecular characterization of the PmrA regulon. J Biol Chem 274: 27185-27190.

13. Tu X, Latifi T, Bougdour A, Gottesman S, Groisman EA (2006) The PhoP/PhoQ two-component system stabilizes the alternative sigma factor RpoS in *Salmonella enterica*. Proc Natl Acad Sci USA 103: 13503-13508.

14. Winfield MD, Latifi T, Groisman EA (2005) Transcriptional regulation of the 4-Amino-4-deoxy-L-arabinose biosynthetic genes in *Yersinia pestis*. J Biol Chem 280: 14765-14772.

15. Hilbert F, Garcia-del Portillo F, Groisman EA (1999) A periplasmic D-alanyl-D-alanine dipeptidase in the gram-negative bacterium *Salmonella enterica*. J Bacteriol 181: 2158-2165.

16. Staggs TM, Perry RD (1991) Identification and cloning of a fur regulatory gene in *Yersinia pestis*. J Bacteriol 173: 417-425.

17. Hanahan D (1983) Studies on transformation of *Escherichia coli* with plasmids. J Mol Biol 166: 557-580.

18. Perez JC, Latifi T, Groisman EA (2008) Overcoming H-NS-mediated transcriptional silencing of horizontally acquired genes by the PhoP and SlyA proteins in *Salmonella enterica*. J Biol Chem 283: 10773-10783.

19. Tabor S, Richardson CC (1985) A bacteriophage T7 RNA polymerase/promoter system for controlled exclusive expression of specific genes. Proc Natl Acad Sci USA 82: 1074-1078.

20. Kalir S, McClure J, Pabbaraju K, Southward C, Ronen M, et al. (2001) Ordering genes in a flagella pathway by analysis of expression kinetics from living bacteria. Science 292: 2080-2083.

21. Cherepanov PP, Wackernagel W (1995) Gene disruption in *Escherichia coli* - Tc^R^ and Km^R^ cassettes with the option of Flp-catalyzed excision of the antibiotic-resistance determinant. Gene 158: 9-14.
